# From Hyperglycemia to Broken Heart Syndrome: A Case of Diabetic Ketoacidosis-Induced Takotsubo Cardiomyopathy

**DOI:** 10.7759/cureus.64907

**Published:** 2024-07-19

**Authors:** Lorena Escaño, Prarthana Desai, Samir Chaudhry

**Affiliations:** 1 Internal Medicine, Danbury Hospital, Danbury, USA

**Keywords:** takotsubo cardiomyopathy (tcm), stress-induced cardiomyopathy, broken heart syndrome, diabetic keto acidosis, diabetes mellitus

## Abstract

Diabetic ketoacidosis (DKA) is one of the hyperglycemic emergencies seen in patients with poorly controlled diabetes mellitus. One of the potential cardiovascular complications of this hyperglycemic crisis, not that well documented in the literature, is takotsubo cardiomyopathy (TCM) also known as stress-induced cardiomyopathy or “broken heart syndrome”. It is a reversible condition where the heart muscle becomes suddenly weakened and stunned, which is mostly known to develop in patients who have suffered a stressful life event or are undergoing an acute illness.

We present an interesting case of a 45-year-old female with a history of poorly controlled diabetes mellitus who presented with significant hyperglycemia and laboratory results concerning DKA. The patient was also complaining of new-onset chest pain on arrival. Further workup revealed elevated troponin, severely reduced ejection fraction, and echocardiographic findings concerning TCM.

The coexistence of DKA and TCM is rare but clinically significant. This case emphasizes the value of clinical vigilance in patients with this hyperglycemic crisis and encourages us to always consider stress-induced cardiomyopathy as a potential complication. Further research is needed to better elucidate the exact mechanisms linking DKA and stress-induced cardiomyopathy. This will help improve outcomes and prevent recurrence in this vulnerable patient population.

## Introduction

Diabetic ketoacidosis (DKA) is one of the metabolic emergencies that can develop in patients with uncontrolled diabetes mellitus. It mostly affects patients with type 1 diabetes but those with poorly controlled long-standing type 2 diabetes are also at risk. The most common precipitating factors include medication non-compliance, new-onset diabetes, acute illness, or infection. As a metabolic emergency, it is not exempt from its own complications. Given its potential to cause significant laboratory abnormalities, when DKA is not diagnosed and managed promptly, it can affect multiple organ systems leading to renal failure, cardiovascular events, cerebral edema, coma, and ultimately death [1-3].

Cardiovascular events are a well-known complication of hyperglycemic crises. However, among these, takotsubo cardiomyopathy (TCM) is less documented in the literature. TCM, also known as stress-induced cardiomyopathy or “broken heart syndrome”, is a reversible cardiac condition in which there is acute transient left ventricular dysfunction with no evidence of obstructive coronary artery disease on imaging. It is characterized by a distinctive ballooning of the left ventricular apex with hypercontractility of the basal segments. Despite resolution of ventricular function usually seen within days to weeks, it has been associated with serious complications such as cardiogenic shock and severe cardiac arrhythmias [4].

TCM has been reported to account for roughly 2-3% of cases of acute coronary syndrome and it is more frequently seen in female patients in the sixth decade of life [5]. The most well-known precipitating factor is an acute emotional or physical stressor. The underlying pathophysiology is still not fully understood, and it is believed to involve a catecholamine surge that ends up stunning the cardiac muscle temporarily [5-7].

A few case reports have documented the occurrence of TCM as a complication of DKA; however, it remains an uncommon and serious clinical scenario with ample room for further investigation [8-10].

## Case presentation

We present a 45-year-old female who presented to the emergency department with new-onset abdominal pain and vomiting. Past medical history was remarkable for hyperlipidemia, managed with rosuvastatin, and a 12-year history of type 2 diabetes mellitus, managed with insulin, with poor medication compliance. 

The patient presented to the hospital reporting diffuse and constant abdominal pain that started two days prior, associated with nausea, multiple episodes of vomiting, and elevated blood glucose reported as “high” on a glucometer. Symptoms persisted and the following day she started to develop left-sided chest pain described as sharp, both at rest and on exertion, non-radiated, and 7/10 in intensity, which prompted her to present to the emergency department for further evaluation. 

On arrival at the emergency department, she was afebrile, hypertensive with a blood pressure of 150/90 mmHg, and tachycardic with a heart rate of 121, respiratory rate of 20, saturating well on room air. Physical examination at the time was only remarkable for tachycardia and mild abdominal discomfort on palpation. 

Laboratory test results on admission are shown in Table 1. Urinalysis showed 4+ ketones and 3+ glucose with no findings suggestive of infection. 

**Table 1:** Pertinent laboratory results on admission

Laboratory test	Laboratory result	Reference value
Glucose	469	70 – 99 mg/dl
Bicarbonate	4	22 – 29 mmol/L
Anion gap	39	10 – 19 mmol/L
Venous PH	6.99	7.32 – 7.43 mmol/L
Beta-hydroxybutyrate	16	0 – 0.4 mmol/L
Lactic acid	6.1	0.5 – 2.2 mmol/L
Creatinine	1.03	0.67 – 1.23 mg/dL
Glomerular filtration rate	83	>60 mL/min
Corrected sodium	148	135 – 145 mmol/L
Potassium	5.4	3.5 – 5.3 mmol/L
White blood cell count	19,000	3,000 – 10,000
Troponin on admission	68	0 – 14 ng/L
Troponin #2	123	0 – 14 ng/L
Troponin #3	167	0 – 14 ng/L
ProBNP	6,228	0 – 299 pg/mL
Hemoglobin A1C	11.2%	<5.7%

**Figure 1:**
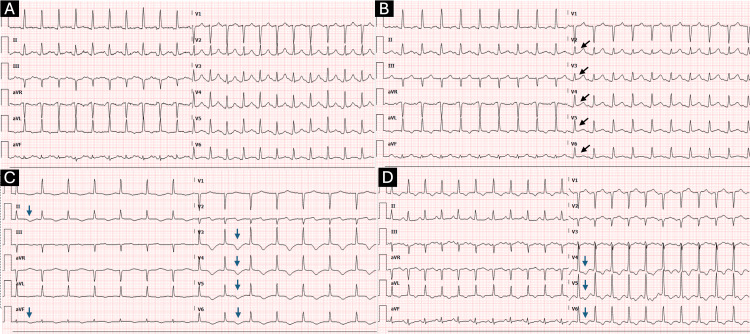
Serial EKGs showing sinus tachycardia, ST-segment elevation more prominent in the anterolateral leads (black arrows), and T wave inversion (blue arrows).

**Figure 2:**
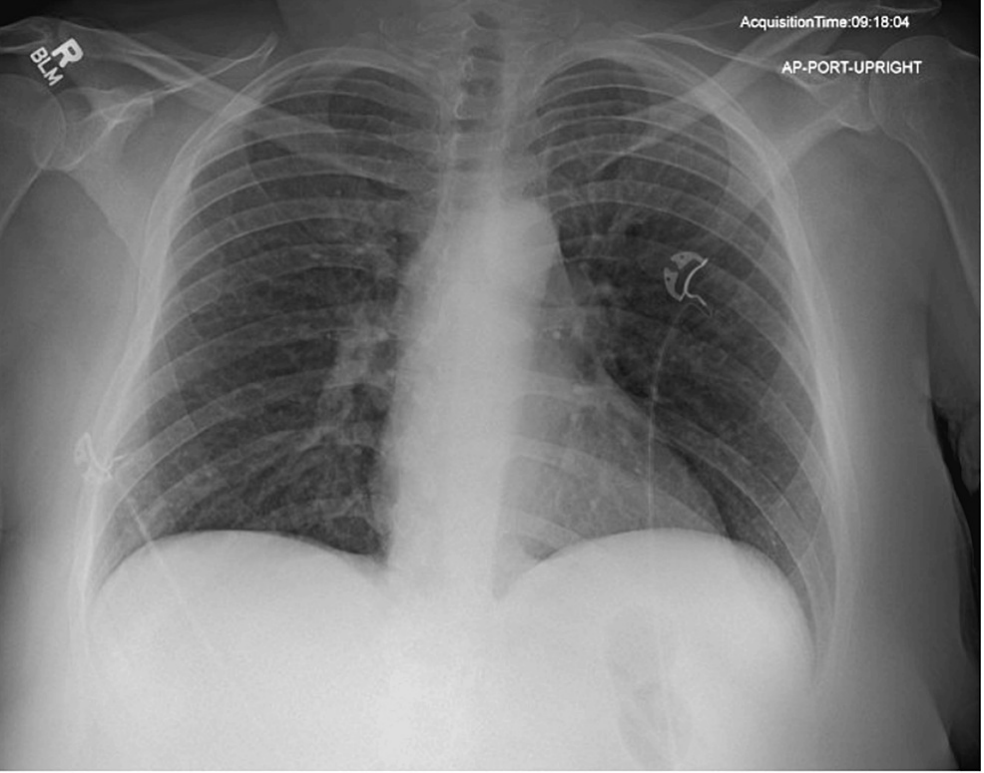
Chest X-ray on admission showing no acute cardiopulmonary process.

**Figure 3:**
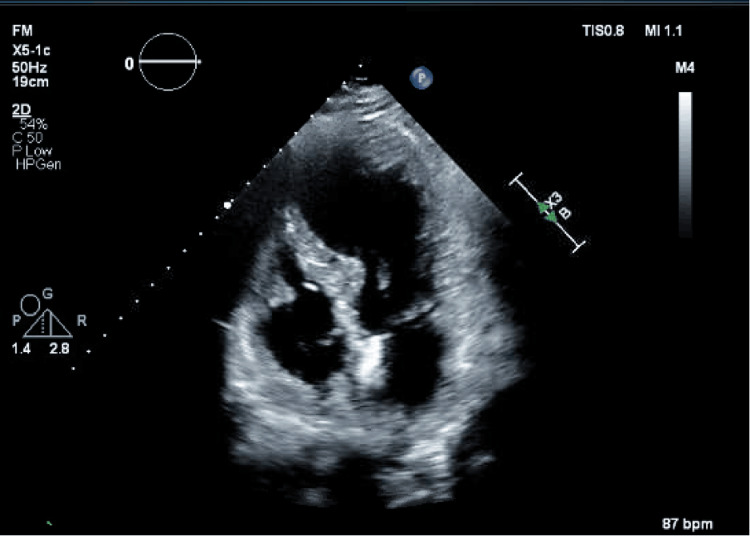
Echocardiogram showing significantly dilated left ventricle with apical ballooning.

Serial EKGs on arrival were remarkable for sinus tachycardia, ST-segment elevation more prominent in the anterolateral leads, and T wave inversion (Figure 1). Chest X-ray was unremarkable for any acute cardiopulmonary process (Figure 2). CT abdomen and pelvis with contrast were also unremarkable. An echocardiogram with an image enhancer showed a significantly dilated left ventricle with a severely reduced ejection fraction of 24%. It also showed akinesis of the mid to distal segments and apical ballooning, with preserved motion of the basal segments (Figure 3).

The patient was admitted to the progressive care unit with a working diagnosis of DKA due to medication non-compliance and concerns about TCM. She was started on insulin drip and bicarbonate, with close monitoring of glucose and electrolytes. Hyperglycemia subsequently improved, metabolic acidosis resolved, and the anion gap closed. Once blood glucose reached 200 mg/dl and oral intake was resumed, she was transitioned to a subcutaneous insulin regimen.

The cardiology team was consulted regarding abnormal EKGs, elevated troponin, and abnormal echocardiogram. The patient underwent left heart catheterization which was unremarkable for obstructive coronary artery disease, reinforcing the diagnosis of stress-induced cardiomyopathy. She was managed conservatively with guideline-directed medical therapy. Chest pain resolved within the first 24 hours. Troponin peaked at 167 and then trended down. She was ultimately discharged home with the plan to follow up with cardiology outpatient. A repeat echocardiogram a month post-hospitalization revealed significant recovery of the left ventricular ejection fraction to 55% with improvement of wall motion abnormalities. 

## Discussion

The diagnosis of TCM can be a challenge and remains a diagnosis of exclusion. This is because patients can present with clinical features that make it difficult to distinguish from acute myocardial infarction, such as ST-segment deviation, elevated troponin, and new wall motion abnormalities. The diagnosis of TCM is made once cardiac catheterization reveals normal or nonobstructive coronary artery disease [5-7]. Our patient presented with new-onset chest pain, elevated troponin, and echocardiogram findings that are considered hallmark features in stress-induced cardiomyopathy. The coronary angiogram with a lack of obstructive coronary artery disease and the recovery of the left ventricular function further reinforced this diagnosis. In addition to this, our patient denied any acute emotional stressors prior to arrival. Infectious workup including imaging and urinalysis was unremarkable. The leukocytosis present on admission was likely reactive and corrected with IV fluids. In view of this, the clinical picture of our patient was more consistent with DKA-induced TCM. 

TCM in the context of DKA remains an uncommon occurrence in the clinical practice and the pathogenesis linking these two entities involves a complex interplay of factors. The pathophysiology of TCM on its own is not fully understood. One theory correlates TCM to heightened activity in the sympathetic nervous system which in turn causes a surplus of catecholamines to be released. This effect is most drastic in the apex of the heart, which harbors a dense concentration of beta-adrenoceptors leading to subsequent failure to contract effectively, which causes the classic reduced movement of the apex pathognomonic in stress cardiomyopathy, as seen in our patient. This is also in part mediated by changes in proteins found in the sarcoplasmic reticulum such as sarcolipin and phospholamban which are crucial in regulating heart contractility. During the acute phase of TCM, a notably high expression of sarcolipin negatively affects the concentration of calcium ATPase (SERCA2), reducing its calcium affinity and calcium-dependent contractile functions [5-7].

One of the most well-known precipitating factors of TCM is an acute emotional stressor; however, its pathogenesis helps us elucidate that any physical stressor capable of inducing that catecholamine surge can be a potential trigger of this cardiovascular event. DKA manifests as pronounced hyperglycemia, increased osmolality, dehydration, and substantial ketoacidosis. These metabolic disturbances arise from both a lack of insulin and a rise in counterregulatory hormones including catecholamines. Apart from this, the acidotic state of DKA characterized by the presence of excessive acetoacetate and beta-hydroxybutyrate further contributes to the disruption in normal sequential chemical processes crucial for myocyte functioning [8-10].

The management of DKA-induced TCM involves addressing metabolic derangements and cardiac dysfunction. Immediate and aggressive treatment of DKA is paramount and it consists of insulin drip and fluid resuscitation with close monitoring of electrolytes. Concurrently, the management of stress-induced cardiomyopathy is supportive and involves guideline-directed medical therapy, which encompasses beta blockers and ACE inhibitors or ARBs, which are beneficial in the management of ventricular dysfunction [5,6]. Our patient had a satisfactory response to the regimen above with significant improvement of ventricular dysfunction one month post-hospitalization. 

The prognosis of TCM is generally favorable, with complete resolution of ventricular dysfunction within weeks to months [5]. However, it is currently uncertain if the overall prognosis and number of recurrences are worse when a diagnosis of uncontrolled diabetes is in the picture [8-10]. A multidisciplinary approach is essential to ensure comprehensive care and optimize outcomes in these patients.

## Conclusions

DKA-induced TCM presents unique diagnostic and therapeutic challenges that require careful consideration in clinical practice. Despite TCM being considered a benign and reversible condition, it is not exempt from complications that can ultimately increase mortality in this already vulnerable patient population.

This case highlights the necessity for further research to deepen our understanding of the pathophysiological mechanisms linking DKA and TCM, and the overall prognosis in affected patients. This will help identify and establish evidence-based diagnostic criteria and treatment protocols that could potentially improve clinical outcomes and reduce recurrence in these patients. 
